# Optical influence of oil droplets on cone photoreceptor sensitivity

**DOI:** 10.1242/jeb.152918

**Published:** 2017-06-01

**Authors:** David Wilby, Nicholas W. Roberts

**Affiliations:** Ecology of Vision Laboratory, School of Biological Sciences, Life Sciences Building, Tyndall Avenue, University of Bristol, Bristol BS8 1TQ, UK

**Keywords:** Oil droplets, Ellipsoid, Optics, Cone, Photoreceptor, Vision

## Abstract

Oil droplets are spherical organelles found in the cone photoreceptors of vertebrates. They are generally assumed to focus incident light into the outer segment, and thereby improve light catch because of the droplets' spherical lens-like shape. However, using full-wave optical simulations of physiologically realistic cone photoreceptors from birds, frogs and turtles, we find that pigmented oil droplets actually drastically reduce the transmission of light into the outer segment integrated across the full visible wavelength range of each species. Only transparent oil droplets improve light catch into the outer segments, and any enhancement is critically dependent on the refractive index, diameter of the oil droplet, and diameter and length of the outer segment. Furthermore, oil droplets are not the only optical elements found in cone inner segments. The ellipsoid, a dense aggregation of mitochondria situated immediately prior to the oil droplet, mitigates the loss of light at the oil droplet surface. We describe a framework for integrating these optical phenomena into simple models of receptor sensitivity, and the relevance of these observations to evolutionary appearance and loss of oil droplets is discussed.

## Abstract (precis)

**Summary:** Optical simulations reveal how both oil droplets and ellipsoid structures in cone photoreceptors can influence optical sensitivity by collecting or reflecting light. Transparent oil droplets and ellipsoids improve sensitivity, whereas pigmented oil droplets reduce sensitivity.

## INTRODUCTION

**Fig. 1.**
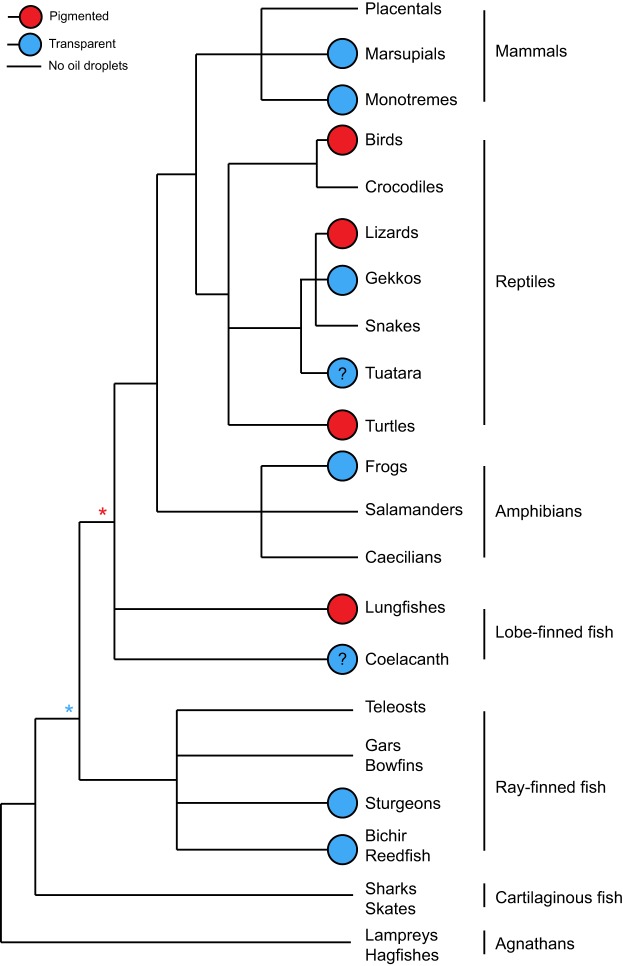
**Summary tree of the pigmentation properties and presence/absence of oil droplets in extant vertebrates.** Blue circles indicate that there are no pigmented oil droplets in any cone of a taxon; red circles show taxa that have at least some pigmented droplets and some transparent, most regularly in the violet-sensitive (VS) and ultraviolet-sensitive (UVS) cones. Asterisks indicate the possible first appearance of pigmented or transparent oil droplets. Question marks indicate uncertainties. Source references for oil droplet traits and more detailed notes are provided in the supplementary information (Table S1). Tree informed by Meyer and Zardoya (2003). Figure courtesy of Olle Lind, Lund University.

The cone photoreceptors of approximately half of the orders of vertebrates contain spherical structures composed of lipids and carotenoid pigment, known as oil droplets (Walls, 1942; Jacobs and Rowe, 2004). Oil droplets are situated immediately prior to the light-sensitive outer segment in the light path, and their role is to influence the light that reaches it. Many oil droplets contain mixtures of carotenoid pigment (Johnston and Hudson, 1976; Toomey et al., 2015) and have been studied predominantly for their spectral filtering properties (e.g. Partridge, 1989; Hart, 2001) and their influence on tuning the spectral sensitivity of colour vision, thereby improving colour discrimination and colour constancy (Vorobyev, 2003). However, the oil droplets of ultraviolet- and violet-sensitive (UVS and VS, respectively) cones are transparent across the visible spectrum (Bowmaker et al., 1997), containing no pigment. Further, numerous species (Fig. 1) only have transparent oil droplets in all of their cone types. Therefore, this widespread presence of transparency indicates that oil droplets must serve a purpose other than just spectral filtering (Walls, 1942; Hart, 2001).

Oil droplets have a relatively high refractive index, a spherical shape and are typically wider than the outer segment (Ives et al., 1983; Young and Martin, 1984; Wilby et al., 2015). Being able to enlarge the area of light capture without increasing the size of the outer segment should, in theory, improve the signal-to-noise ratio and reduce energetic cost (Ives et al., 1983; Young and Martin, 1984; Stavenga and Wilts, 2014). Nevertheless, the extent to which oil droplets improve light capture is still unclear. Prior efforts have proposed that oil droplets gather more light into the outer segment (Govardovskiì et al*.,* 1981; Ives et al., 1983; Young and Martin, 1984; Stavenga and Wilts, 2014). However, this might not be the case for all oil droplets, with a recent study discovering that the pigmented droplets in simulations of cone photoreceptors in the chicken (*Gallus gallus domesticus*) reduced transmission of light into the outer segment for the regions of the spectrum to which the visual pigments were sensitive (Wilby et al., 2015). Only the transparent oil droplet of the violet cone increased light transmission into the outer segment by approximately 50%.

This raises the questions: what properties of oil droplets influence light catch?; and how do oil droplets perform relative to the ‘ideal’ light-coupling scenario in which all incident light within the area of the oil droplet is focused into the outer segment?

In this study, we use numerical optical calculations informed by morphological and optical measurements to investigate the influence oil droplets have on optical power in the outer segment. We include the optics of the outer segment, which act as a waveguide to confine light to the regions of the retina containing the light-sensitive pigment. Finally, we examine the optical role of the ellipsoid in conjunction with the oil droplet in the concentration of light into the outer segment.

## MATERIALS AND METHODS

### Optical simulations

Calculations were performed with the freely available finite-difference time-domain (FDTD) simulation software, MEEP (MPI version 1.2; Oskooi et al., 2010), using the computational facilities of the Advanced Computing Research Centre, University of Bristol. Simulations took advantage of the rotational symmetry of the models about the *z*-axis, and computations were performed in cylindrical polar coordinates for a thin wedge of the model (see Fig. S1). Carotenoid absorption spectra along with refractive index measurements were used to model the dielectric function of the oil droplets as previously described (Wilby et al., 2015).

For models of oil droplets based on the three species in this study [chicken, *Gallus gallus domesticus* (Linnaeus 1758); red-eared slider, *Trachemys scripta elegansí* (Wied-Neuwied 1839); and African clawed frog, *Xenopus laevis* Daudin 1802], calculations were performed for specific oil droplet and outer segment dimensions. Dimensions and refractive indices were taken from the literature and are summarised in Table S2. For simplicity, a wavelength-invariant value of refractive index of 1.45 was used for the outer segment, as measured previously (Wilby et al., 2015). The refractive index change of the lipid membrane across the visible spectrum is <0.01 (Roberts et al., 2009) and the visual pigment has minimal influence on the refractive index (Stavenga and van Barneveld, 1975). Analyses of the sensitivity of the models to the refractive index of the outer segment demonstrated that a value of 1.45 is a conservative choice. The surrounding medium was given the refractive index 1.35, as calculated by Enoch and Tobey (1978). Simulations that incorporated the ellipsoid modelled it as a cylinder preceding the oil droplet and surrounding its front hemisphere, similarly to Wilby et al. (2015). Ellipsoids were equal in radius to the oil droplet, had a refractive index of 1.43 and were 3.5 μm long. Full details of all simulation parameters are given in Table S2.

Following Ives et al. (1983), we define the volume-averaged enhancement factor, *D*, as the ratio of the integral of **E***.*E* (as a proxy for light intensity) within the outer segment in the presence of an oil droplet (OD) to the integrated electric field intensity in the absence of the oil droplet (NOD):
(1)
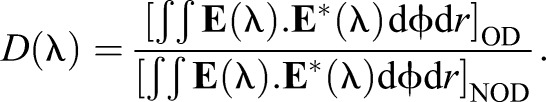


Integrals were performed numerically in cylindrical polar coordinates over the volume of the outer segment (which effectively reduces to an area under cylindrical symmetry). The electric field vector is composed of components [*E*_r_,*E*_φ_,*E*_z_] along the radial, polar and *z* (propagation) axes, respectively. The complex conjugate is indicated by *.

Additionally, we define an enhancement as predicted using the geometry alone, *D*_G_, as the ratio of the cross-sectional areas of the oil droplet and the outer segment, which reduces to:
(2)
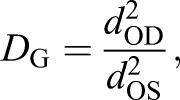


where *d*_OD_ and *d*_OS_ are the diameter of the oil droplet and the outer segment closest to the oil droplet, respectively. This is analogous to the use of the oil droplet diameter as the photoreceptor diameter in typical photoreceptor sensitivity calculations (e.g. Land, 1981; Warrant and Nilsson, 1998). We also define the fraction, *F*, of light arriving within the cross-sectional area of the oil droplet that upon focusing arrives in the outer segment as:
(3)
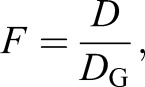


which can be incorporated into existing models of photoreceptor sensitivity (e.g. Land, 1981; Warrant and Nilsson, 1998) as a multiplicative factor that accounts for losses and gains due to optical phenomena (Olsson et al., 2017).

### Measurement of oil droplet refractive index

*Xenopus laevis* adults were culled by an overdose of anaesthetic (MS222) and destruction of the central nervous system according to the ethical guidelines of the University of Bristol. Eyes were enucleated from two animals, hemisected and the retina removed. Pieces of retina approximately 2×2 mm^2^ were separated by repeated pipetting in deionised water and centrifuged at 13,137 ***g*** for 2 min.

For *X. laevis* oil droplets, refractive indices were measured for this study using a commercial digital holographic microscope (DHM; T1000; LyncéeTec, Lausanne, Switzerland). Measurements were made at free-space wavelengths of 445, 488, 515 and 640 nm. The refractive index measurement method of Schürmann et al. (2015) was implemented using a combination of proprietary DHM software, Koala (LyncéeTec) and bespoke code written in MATLAB (v8.3; MathWorks, Natick, MA, USA).

The two-term Cauchy equation:
(4)
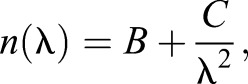


where *n* is the refractive index as a function of free-space wavelength, λ, and *B* and *C* are the Cauchy coefficients, was fitted to the discrete measurements to calculate wavelength dependence of the refractive index of *X. laevis* oil droplets. The Cauchy equation, in comparison to the more physically descriptive Sellmeier equation, is accurate in the wavelength range used here for normally dispersive materials and is somewhat simpler (Born and Wolf, 1999; Leertouwer et al., 2011).

Oil droplet refractive indices and absorbance spectra (as a function of wavelength) for *G. g. domesticus* were taken from Wilby et al. (2015); and for *T. scripta elegans* from Ives et al. (1983), Liebman and Granda (1971) and Strother (1963).

### Ethical approval

All applicable international, national, and/or institutional guidelines for the care and use of animals were followed.

## RESULTS

First, we present the results of how variation in the geometry and refractive index of the oil droplets affect the light catch in model outer segments. We then use the models of cone photoreceptors from *G. g. domesticus*, *T. s. elegans* and *X. laevis* that are based on geometrical and optical measurements of real photoreceptor cells to investigate how oil droplet pigments affect optical enhancement and absorption. Lastly, we show how the ellipsoid of chicken photoreceptors plays a role in light catch enhancement.

### Higher oil droplet refractive index decreases light catch

**Fig. 2.**
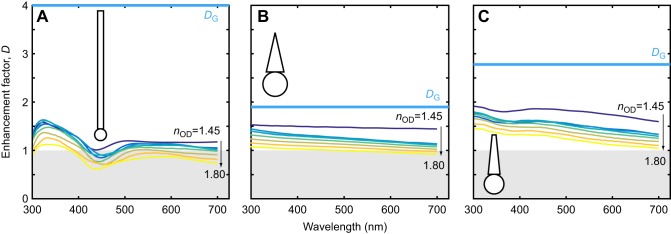
**Simulated enhancement factor curves for three model photoreceptors.** Based on the dimensions of cones in (A) birds (*l*_OS_=30 μm, *d*_OS_=1.5 μm, *d*_OD_=3 μm), (B) frogs (*l*_OS_=12 μm, tapering *d*_OS_=4.5–0 μm, *d*_OD_=6.2 μm) and (C) turtles (*l*_OS_=10 μm, tapering *d*_OS_=3–1 μm, *d*_OD_=5 μm), where *l*_OS_ is the outer segment length, and *d*_OS_ and *d*_OD_ are the diameters of the outer segment and the oil droplet, respectively. Families of curves were calculated for a wavelength-invariant value of the refractive index of the oil droplet, *n*_OD_, increasing in steps of 0.05 from 1.45 to 1.8. Grey regions show enhancement factors <1, corresponding to loss of light because of the oil droplet. Thick light blue lines show *D*_G_. In all three cases, the higher the refractive index of the oil droplet, the lower the enhancement factor. In no case did the enhancement factor approach *D*_G_.

In simulations of transparent oil droplets before cylindrical, conical and truncated-conical outer segments, the enhancement factor decreased as the refractive index of the oil droplet, *n*_OD_, increased (Fig. 2). Moreover, the enhancement factors do not reach the ideal predicted using receptor geometry, *D*_G_. In cylindrical outer segments, similar to chicken cones (*d*_OD_=3 μm; *d*_OS_=1.5 μm; *l*_OS_=30 μm; Wilby et al., 2015) *D*_G_ is 4, but calculated enhancement factors vary between 0.6 and 1.7, with the lowest values occurring for higher *n*_OD_ and longer wavelength (Fig. 2A). For the conical outer segment model, similar to amphibian cones (*d*_OD_=3.1 μm; *d*_OS_=2.25 μm; *l*_OS_=12 μm; Röhlich and Szél, 2000), enhancement factors are predominantly >1 but again do not reach the *D*_G_ of 1.90 (Fig. 2B). Similarly in the truncated cone model, similar to turtle cones (*d*_OD_=2.5 μm; *d*_OS_=1.5-0.5 μm; *l*_OS_=10 μm; Ives et al., 1983), enhancement factors are >1 but do not reach the *D*_G_ of 2.78 (Fig. 2C).

### Oil droplets enhance light catch more for shorter outer segments

**Fig. 3.**
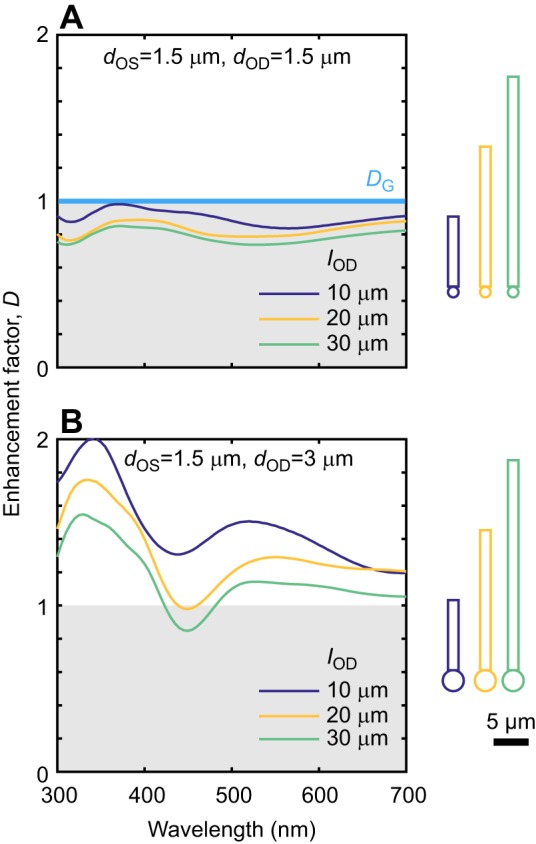
**Simulated enhancement factors for increasing outer segment length, *l*_OS_.** *d*_OS_=1.5 μm and *n*_OD_=1.5. Grey regions indicate oil droplets resulting in loss of light. (A) *d*_OD_=1.5 μm. (B) *d*_OD_=3 μm. Thick light blue line indicates *D*_G_ of 1 in A and 4 in B, falling outside the axis limits. Enhancement is greater for shorter *l*_OS_ for both values of *d*_OD_.

We created sets of simulations with varying *l*_OS_ for cylindrical outer segments with *d*_OS_=1.5 μm and oil droplets of the same or double the diameter. In both cases, enhancement factors were larger for shorter outer segments (Fig. 3). This effect, however, is relatively small over the range of outer segment lengths found in nature, as the values of *l*_OS_ used here were 10, 20 and 30 μm. For oil droplets of the same diameter as the outer segment (Fig. 3A), enhancement factors were <*D*_G_ =1. For oil droplets double the diameter (Fig. 3B), enhancement factors never approached the *D*_G_ of 4.

### Greater enhancement for larger oil droplets and wider outer segments

**Fig. 4.**
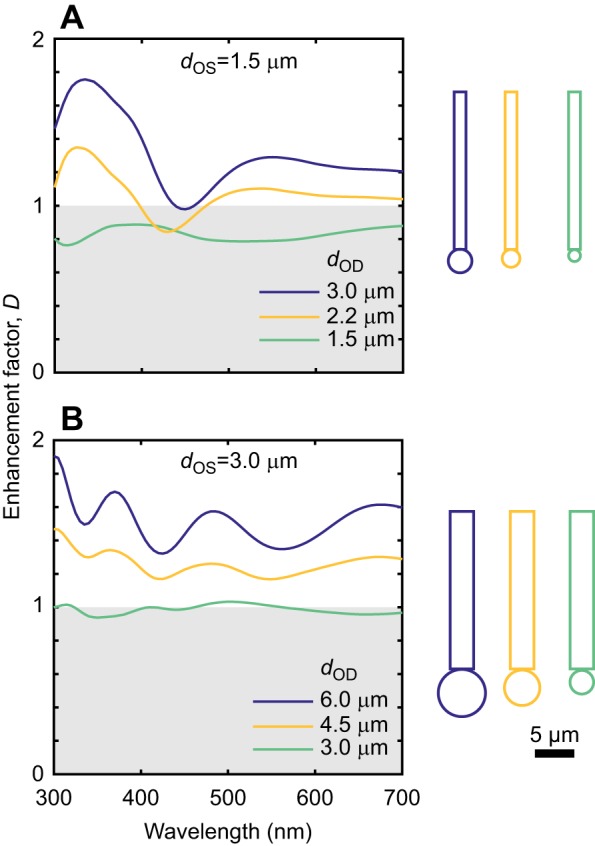
**Simulated enhancement factors for increasing sizes of oil droplet.** *l*_OS_=30 μm and *n*_OD_=1.5. Grey regions indicate oil droplets resulting in loss of light. Larger oil droplets result in greater enhancement factors. Wider outer segments and larger oil droplets give larger enhancement factors.

In sets of simulations for constant dimensions of the outer segment but increasingly large oil droplets, enhancement was greater for larger oil droplets (Fig. 4). For *d*_OD_=*d*_OS_, enhancement factors approached the *D*_G_ of 1. For *d*_OS_=3 μm, enhancement was very close to 1 and even slightly greater for some wavelengths – the only scenario tested here in which the simulated enhancement factor exceeded the geometrically predicted value. For larger oil droplets, enhancement was increased above 1, but did not approach the *D*_G_ of 4. The simulation sets presented in Fig. 4 illustrate examples for which the geometrically predicted values are the same but have differing dimensions. For instance, the geometrically predicted values for the solid lines in Fig. 4 have an equal value of 4 but have largely differing enhancement factor curves, with the greater values occurring for larger *d*_OS_ and *d*_OD_.

### Refractive index of *Xenopus laevis* oil droplets

**Fig. 5.**
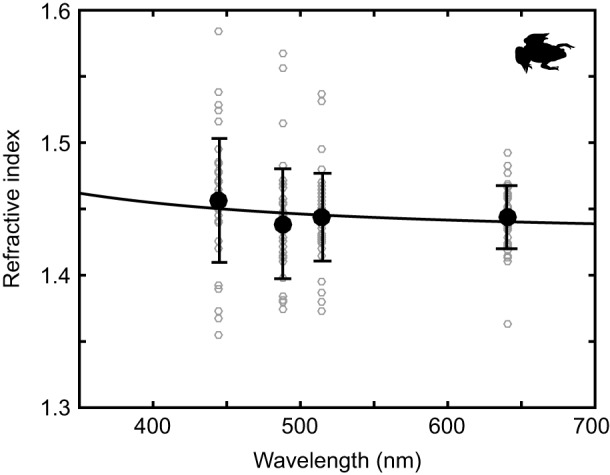
**Refractive index of *Xenopus laevis* oil droplets measured by digital holographic microscopy.** Grey open circles show individual measurements. Black filled circles show mean values at each wavelength with error bars showing single standard deviations. Line shows the Cauchy equation fit to the data points. *Xenopus* silhouette modified from photograph by Brian Gratwicke available on flickr under Creative Commons attribution license.

In order to create an optical simulation for the cones of *X. laevis*, which have only transparent oil droplets, we first had to measure the oil droplet refractive index as a function of wavelength. The refractive index measurement method resulted in large variance, though normally distributed (Fig. 5). The two-term Cauchy equation (Eqn 4) was fit to these measurements to calculate the refractive index of the oil droplets as a function of wavelength. The Cauchy coefficients for the fit were *B*=1.4311 and *C*=3.8×10^3^ nm^2^. In addition, we found no evidence of more than one population of oil droplets with respect to their refractive index, indicating that the oil droplets in different classes of cone in *X. laevis* share similar optical properties (see Fig. S2). As is typical of non-absorbing materials, the dispersion is weak across the 350–700 nm wavelength range (Born and Wolf, 1999).

### Pigmented droplets reduce light catch for relevant wavelengths

**Fig. 6.**
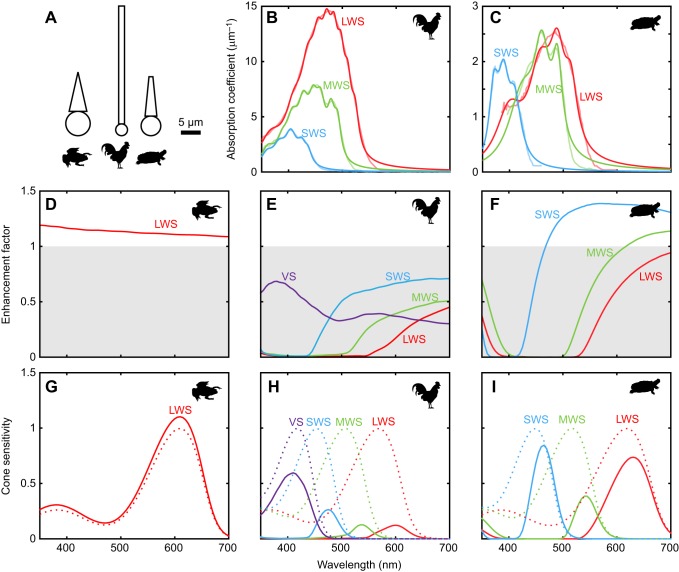
**Influence of pigmented and unpigmented oil droplets on sensitivity of cones in *Xenopus laevis*, *Gallus gallus domesticus* and *Trachemys scripta elegans* in the absence of the ellipsoid.** (A) Relative cone photoreceptor dimensions used in calculations. (B,C) Absorption coefficients of the long-, medium- and short-wavelength-sensitive (LWS, MWS and SWS, respectively) cone oil droplets in the chicken and turtle, respectively. Pale lines show measured spectra, dark lines show modelled spectra. (D–F) Oil droplet enhancement factors for the cone photoreceptors of the three species. (G–I) Relative cone sensitivities using the calculated enhancement factors. Dotted lines show the visual pigment absorbance templates. Solid lines show the result of multiplying the normalised visual pigment absorbance by the enhancement factor. (D,G) *Xenopus laevis*. (B,E,H) *Gallus gallus domesticus*. (C,F,I) *Trachemys scripta elegans*. The chicken silhouette was designed by freepik. *Xenopus* and turtle silhouettes were modified from photographs by Brian Gratwicke and Jim Capaldi made available on Flickr under Creative Commons attribution licenses.

Enhancement factors were calculated for oil droplet models with refractive indices, dimensions and absorption spectra for cone photoreceptors of *X. laevis*, *G. g. domesticus* and *T. s. elegans* (Fig. 6A–C). Enhancement factors for the pigmented droplets of chicken and turtles generally reflected those for transparent droplets in regions of the spectrum for which there was low absorption of light in the oil droplet. Predictably, low values of enhancement were seen where absorption was strong (Fig. 6B,C,E,F). The transparent droplet of *X. laevis* has an enhancement factor >1 covering the entire spectrum, universally increasing light catch (Fig. 6D). However, for regions of the spectrum for which the visual pigments are sensitive, and particularly at the wavelength of peak visual pigment sensitivity (λ_max_) of the cones, pigmented droplets mostly reduce light catch and hence cone sensitivity (Fig. 6E,F,H,I). Oil droplets in chicken photoreceptors are all predicted to have enhancement factors <1 (i.e. reduce light catch, Fig. 6E,H). Those in turtle photoreceptors, while having enhancement factors >1 for longer wavelengths, have values <1 at the λ_max_ of all three cone types (Fig. 6I).

### Ellipsoids improve enhancement

**Fig. 7.**
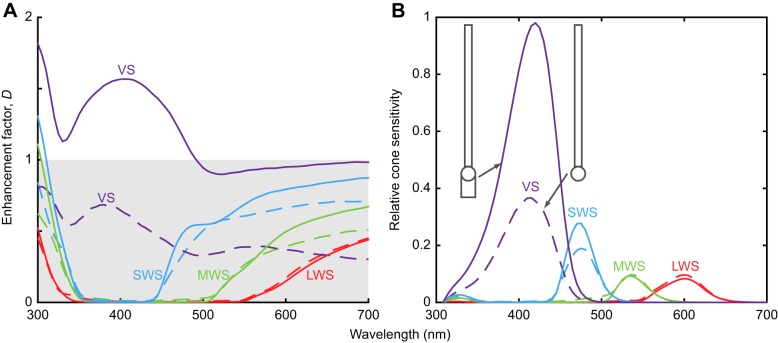
**Impact of the ellipsoid on enhancement factor and relative sensitivity for chicken cone photoreceptors.** (A) Enhancement factors with and without the ellipsoid. Dashed lines show enhancement factors without the ellipsoid; solid lines show enhancement factors including both ellipsoid and oil droplet. Greatest increase is seen for the violet-sensitive (VS) cone, which with the addition of an ellipsoid has an enhancement factor much larger than 1. In the SWS and MWS receptors, a small increase is seen. In the LWS receptor, a very slight decrease in enhancement is seen for the visible spectrum. (B) Relative sensitivity of chicken cones with and without the ellipsoid. Ocular media transmittance is included as measured by Lind and Kelber (2009b).

Currently, *G. g. domesticus* is the only species for which all the requisite optical and structural measurements of the ellipsoid are available (Wilby et al., 2015). The modelling data demonstrated that in this species, the ellipsoid has a substantial effect on the optics and the addition of the ellipsoid in the VS, short-wavelength-sensitive (SWS) and medium-wavelength-sensitive (MWS) cones increased the enhancement factor (Fig. 7A). The greatest enhancement was seen for the VS cone, for which the enhancement is approximately doubled on addition of the ellipsoid. Enhancement also becomes almost entirely >1 for wavelengths to which the VS cone is sensitive (Fig. 7B).

It is clear that the ellipsoid increases the enhancement within VS, SWS and MWS chicken cones, particularly the transparent VS cone, and it is logical to assume that similar optical effects take place for the ellipsoids of cones in other species with both transparent and pigmented oil droplets. This leads to the conclusion that the enhancements shown in Fig. 6 for *X. laevis* and *T. s. elegans* are likely to underestimate real enhancement, similar to the case for the chicken.

## DISCUSSION

The extent of enhancement in light catch provided by an oil droplet is profoundly variable across different types of cone as well as across the visible spectrum. We have shown that several factors combine to influence the light coupling into the outer segment. These include, but are not limited to: the oil droplet refractive index; dimensions of the oil droplet and outer segment; and the presence and refractive index of the ellipsoid, which are discussed below.

### Cone structure and oil droplet refractive index

We have calculated the change in light intensity in the outer segment that is due to the optical influence of transparent oil droplets in order to investigate the effects of cone refractive index and geometry on the passage of light and hence sensitivity. The following three findings summarise the combined effects of these changes.

First, an increase in refractive index compromises the ability of the oil droplet to concentrate light into the outer segment. This occurs regardless of photoreceptor structure (Fig. 2) and has been shown previously for pigmented droplets (Ives et al., 1983; Wilby et al., 2015). A potential source for this reduction in enhancement may be due to increased reflectivity from the oil droplet interface for higher values of *n*_OD_ (Wilby et al., 2015).

Second, three observations show that receptor geometry can affect the extent to which the oil droplet enhances light capture: shorter outer segments may benefit from greater enhancement (Fig. 3); larger oil droplets capture light over a larger area and so collect more light into the outer segment, giving greater enhancement (Fig. 4); and wider outer segments benefit from greater enhancement (Fig. 4). The root of this last observation lies in waveguide phenomena – a wider outer segment will support a greater number of waveguide modes (Snyder and Love, 1983; Stavenga, 2003).

And third, geometrical models of photoreceptor sensitivity (Land, 1981; Warrant and Nilsson, 1998) are not designed to take wave optics into account. The normal assumption is that oil droplets focus all light within their cross-sectional areas into the outer segment (e.g. Lind and Kelber, 2009a). Here, we have seen that this is not the case, and when optical effects are incorporated, *F*<1. The implication is that calculated absolute sensitivities of photoreceptors with oil droplets will be reduced if optical effects are included.

One factor that may hinder the ability of oil droplets to enhance light capture is their position relative to the outer segment; oil droplets are in direct contact with the outer segment aperture. Man-made ball lens–waveguide assemblies are an equivalent synthetic system used to couple light from light sources into dielectric waveguides. Here, the highest coupling efficiencies in these systems are seen for intermediate distances between the ball lens and waveguide entrance (Ratowsky et al., 1997), and although operating on a larger scale, similar optical considerations apply.

The curves in Figs 2–4 are non-trivial functions of wavelength, displaying local minima and varying behaviour depending upon the photoreceptor structure. This is due to the relative prominence of the influence of contributing optical phenomena including Mie scattering and waveguidance, which have contrasting wavelength dependencies. For instance, the wavelength position of peaks and troughs in the enhancement factor curves correspond with Mie scattering behaviour, such as that predicted by the anomalous diffraction approximation (van de Hulst, 1981). Differing combinations of refractive index and oil droplet diameter result in a Mie scattering efficiency curve that oscillates as a function of wavelength at the length scales seen here. In contrast, power contained in a dielectric waveguide decreases gradually with increasing wavelength (Snyder and Love, 1983). Both of these phenomena play a role in governing the sensitivity of oil-droplet-bearing photoreceptors. By using FDTD simulation, a full-wave optical approach, all classical optical effects are included in the solution of Maxwell's equations.

### Pigmented oil droplets

None of the pigmented droplets examined here increased light capture around the peak absorbance of the visual pigment (Fig. 7). This tells a very different story to the general assumption that all light within the inner segment is focused by the oil droplet into the outer segment. It is only the transparent oil droplets that consistently exhibit the increased enhancement factors. The ellipsoid does seem to generally increase the enhancement factors, particularly in the VS cones of *G. g. domesticus*, which, in the presence of the ellipsoid, increases light capture by 50%. This essentially justifies the retention of transparent oil droplets in VS/UVS cones, meaning that they improve signal-to-noise ratio in these cones. Pigmented droplets, separately, tune spectral sensitivity in the other cone types, but do not help with light capture. This effect of the ellipsoid increasing on-axis transmission of light into the outer segment is consistent with earlier observations (Govardovskiì et al., 1981; Wilby et al., 2015).

### Absolute sensitivity

The prediction from the calculations presented here is that oil droplets do not collect as much light as geometrical calculations would predict. Therefore, the expectation is that oil-droplet-bearing cones are not as sensitive to light as previously thought. However, this is not a straightforward prediction to test. In a recent experiment, Olsson et al. (2017) performed behavioural tests of the intensity thresholds in a colour discrimination task in chickens. Discriminability was modelled using the receptor noise limited model in which cone quantum catches were calculated using both geometrical considerations and the optical simulation approach presented here. The optical simulation sensitivity models that incorporated wave-optical effects predicted the number of photoreceptors required an order of magnitude more accurately than those relying on geometrical calculations. This demonstrates, to some extent, that wave-optical phenomena in oil droplet-bearing cones do indeed impact absolute sensitivity of photoreceptors.

Further, absolute sensitivity is also governed by the angular sensitivity of a photoreceptor (as well as the *f*-number of the eye). Here, we have concentrated on light propagating parallel to the photoreceptor axis, whereas previous studies have observed the effect of certain oil droplets on the angular sensitivity of cone photoreceptors (Govardovskiì et al., 1981; Wilby et al., 2015). Both studies found that oil droplets narrow the angular sensitivity; Govardovskiì et al. (1981) explain that though the oil droplet increases the quantum catch for on-axis propagation of light, because of the narrowing of angular sensitivity, it results in no overall greater sensitivity.

### Outer segments in enhancement calculations

Ives et al. (1983) reported enhancement factors in the cone of the turtle *T. s. elegans* of 2–4 for the clear, yellow and red oil droplets corresponding to the SWS, MWS and LWS cones, respectively. However, in our calculations, we find no enhancement factors >2 for any of the experimentally valid properties used in our calculations. Importantly, the calculations of Ives et al. (1983) only used an analytical Mie scattering approach, and therefore by definition did not include the optical properties and structure of the outer segment itself in their models. In an attempt to reconcile the differences between these two sets of calculations, we performed simulations using similar properties to those used in Ives et al. (1983) with and without the outer segment. Our results (see Fig. S3) show that without the outer segment (i.e. the oil droplet is isolated in isotropic material of a single refractive index), enhancement factors appear to greatly exceed 2 at wavelengths where there is little absorption in the oil droplet. Overall, our results show that it is essential to include both the outer segment and ellipsoid in any optical model of photoreceptors.

### Optics and spectral sensitivity

One noticeable effect is that the enhancement factor is wavelength dependent. This may lead to modulation of the receptor sensitivity via the alteration of the relative abundance of photons of certain wavelengths within the outer segment. It has previously been shown that real photoreceptor spectral sensitivity is altered by waveguide effects. Due to the wavelength dependency of guided power, there is greater sensitivity to shorter wavelengths in the photoreceptors of the small white butterfly (Stavenga and Arikawa, 2011). It remains to be seen whether scattering effects such as those investigated here alter the relative spectral sensitivity of vertebrate photoreceptors with any measurable significance. Models of spectral sensitivity that only include absorption have been relatively accurate so far in explaining colour vision, in birds for instance (Vorobyev and Osorio, 1998).

### Evolutionary loss of oil droplets

Throughout vertebrate evolution, it is unclear whether oil droplets first appeared in the transparent or pigmented form; moreover, they may also have switched between states more than once. It also seems that because oil droplets have been lost from various major lineages, they are not always advantageous (Robinson, 1994; Rowe, 2000; Jacobs and Rowe, 2004). Oil droplets do not seem capable of improving light capture and tuning spectral sensitivity by filtering at the same time. Under the previous dogma that all oil droplets improve light capture in cones, there seems to be little disadvantage to their presence in the retina. However, we see here that oil droplets must be relatively large and transparent in order to substantially improve light capture. On increasing the size of the oil droplet, fewer receptors can be packed into a certain area of the retina, thus reducing spatial acuity. The transparent oil droplet is essentially a device to improve the signal-to-noise ratio of the cone mechanism, and therefore this does come at the expense of visual acuity to a certain extent. We suggest that if a better tool for improving signal-to-noise ratio evolved that did not sacrifice acuity, this would be grounds for abandoning the transparent oil droplet. Such a feature might be represented by a less noisy visual pigment or perhaps a dynamic spatial pooling mechanism.

### Conclusions

We find that optical enhancement provided by oil droplets is highly variable with receptor morphology and refractive index as well as wavelength. Our primary conclusion is that oil droplets almost never collect as much light as predicted by purely geometrical considerations. In general, transparent oil droplets increase the light collected into the outer segment and pigmented droplets decrease collection for wavelengths around the maximal visual pigment sensitivity. Ellipsoids in avian cones act to alleviate the light loss, resulting in an overall gain in light capture in the VS cone. The ultimate implication for vision in oil droplet-bearing cones is that absolute receptor sensitivity is largely reduced in comparison to models that do not include optical phenomena.
